# Reflections on amyloidosis in Papua New Guinea

**DOI:** 10.1098/rstb.2008.0073

**Published:** 2008-11-27

**Authors:** Per Westermark, Gunilla T. Westermark

**Affiliations:** 1 Department of Genetics and Pathology, Uppsala UniversitySE 751 85 Uppsala, Sweden; 2 Department of Clinical and Molecular Medicine, Linköping UniversitySE 581 85 Linköping, Sweden

**Keywords:** amyloid, seeding, transmission, protein AA

## Abstract

The amyloidoses comprise a heterogeneous group of diseases in which 1 out of more than 25 human proteins aggregates into characteristic beta-sheet fibrils with some unique properties. Aggregation is nucleation dependent. Among the known amyloid-forming constituents is the prion protein, well known for its ability to transmit misfolding and disease from one individual to another. There is increasing evidence that other amyloid forms also may be transmissible but only if certain prerequisites are fulfilled. One of these forms is systemic AA-amyloidosis in which an acute-phase reactant, serum AA, is over-expressed and, possibly after cleavage, aggregates into amyloid fibrils, causing disease. In a mouse model, this disorder can easily be transmitted from one animal to another both by intravenous and oral routes. Also, synthetic amyloid-like fibrils made from defined small peptides have this property, indicating a prion-like transmission mechanism. Even some fibrils occurring in the environment can transmit AA-amyloidosis in the murine model. AA-amyloidosis is particularly common in certain areas of Papua New Guinea, probably due to the endemicity of malaria and perhaps genetic predisposition. Now, when kuru is disappearing, more interest should be focused on the potentially lethal systemic AA-amyloidosis.

## 1. Introduction

It has been increasingly acknowledged that misfolding and aggregation of proteins into what we call amyloid is a very common phenomenon and associated with several different disorders. Amyloid is a generic name for *in vivo* formed fibrillar deposits, characteristic of a number of diseases and which have in common misfolding of a protein with a high degree of beta-structure and which then aggregate into typical fibrils of undetermined length. The fibrils are remarkably similar in spite of very varying protein nature. Typical of the fibrils is their resistance to degradation and adoption of particular binding properties, e.g. to different dyes such as thioflavine T and Congo red. Amyloid is thus a heterogeneous substance and in humans more than 25 different proteins are known to occur in this form, each typical of one or a few clinical conditions (Westermark *et al*. 2007). The amyloid can either be found in one tissue (local) or in many organs around the body (systemic). The local forms are most common and often found in the central nervous system, the cardiovascular system or in some endocrine organs.

## 2. Amyloidosis in Papua New Guinea

Deposits of amyloid occur in prion diseases including kuru (Zigas & Gajdusek 1959) and are typical of some common maladies such as Alzheimer's disease and type 2 diabetes. These are two disorders expected to increase rapidly in Papua New Guinea when life expectancy increases and a western lifestyle is adopted (Hodge *et al*. 1995). Consequently, problems concerning pathogenesis and importance of amyloid are highly relevant also to Papua New Guinea.

The systemic amyloidoses comprise a heterogeneous group of disorders in which amyloid deposits are formed from a circulating precursor, usually synthesized in one tissue but giving rise to deposits in many organs of the body. At least 12 different proteins have this property and each is characteristic of one or a few clinical syndromes. Most systemic amyloidoses are life-threatening disorders. One of the most common is reactive (secondary or AA-) amyloidosis. The fibril is derived from the acute-phase reactant apolipoprotein serum AA (SAA), whose expression in the liver is under the control of interleukin (IL)-1, IL-6 and tumour necrosis factor (TNF)-alpha. AA-amyloidosis is thereby a consequence of some chronic inflammatory disorders, notably infectious diseases, such as tuberculosis and leprosy, and rheumatic inflammations, particularly rheumatoid arthritis. The major symptoms in AA-amyloidosis come from the kidneys.

Systemic AA-amyloidosis occurs worldwide but has been reported to be particularly prevalent in certain areas of Papua New Guinea (McAdam *et al*. 1996). Cooke & Champness (1970) reported an autopsy series of 1101 individuals with a prevalence of 7.3 per cent of systemic amyloidosis, which is uniquely high. At that time, determination of type was uncertain and their conclusion that approximately 50 per cent of these cases were of ‘primary’ type was probably wrong. Most probably the majority of the patients had AA-amyloidosis. In order to explain the high prevalence, these authors suggested that some hereditary factor may be important in the pathogenesis. AA-amyloidosis in Papua New Guinea is not rare in children, in whom a characteristic hallmark may be amyloid goitre combined with renal symptoms, an uncommon combination elsewhere (Cooke & Champness 1972).

Cooke & Champness could not find a cause of amyloidosis such as leprosy or tuberculosis in approximately 50 per cent of their cases. At that time, malaria was not regarded as a possible cause. It became apparent that AA-amyloidosis was very prevalent among the Anga people in the upper Watut Valley but much more uncommon in the same linguistic group in the highland area near Aseki (see McAdam *et al*. 1980). A main difference between these two groups of people was the very high prevalence of malaria with tropical splenomegaly syndrome in the Watut Valley (McAdam 1976). McAdam used the newly developed subcutaneous fat needle biopsy (Westermark & Stenkvist 1973) for a unique point prevalence study and found 8 with amyloidosis among 76 (10.5%) biopsied individuals in the Watut Valley area but none out of 72 among the highlanders (McAdam *et al*. 1980). At that time, the AA-nature of this amyloidosis had been shown (Anders *et al*. 1976). Again, malaria was suspected to be a main cause of the amyloid, although some hereditary factor was suspected to be important as well.

**Figure 1:**
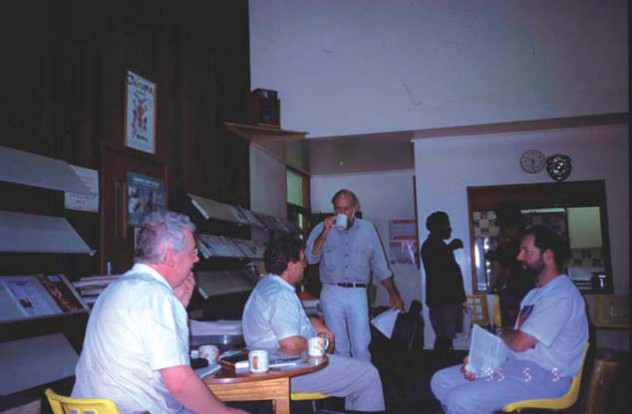
At the Papua New Guinea Institute of Medical Research, Goroka, 1995. In the middle are Dr McAdam and Dr Alpers.

In 1995, we performed a research tour, led by Dr Keith P. W. J. McAdam, to Goroka (figure 1) and to Watut Valley, Papua New Guinea, for further studies of AA-amyloidosis, particularly regarding possible hereditary factors. Although not all objectives were reached, we identified, by protein sequence analysis of purified protein AA from one patient, a previously undescribed subspecies, derived from an SAA variant that was designated SAA1δ (Westermark *et al*. 1996). Human acute-phase SAA is expressed by two different genes, SAA1 and SAA2, and both occur with allelic variants. SAA1α is most commonly found in amyloid fibrils in Caucasians and Asiatic people. In mice, two closely related SAA allelic forms occur but only one forms fibrils and, hypothetically, the human SAA1δ may be more amyloidogenic than other variants, making individuals who express this type more susceptible to develop amyloidosis. Direct biochemical proof is still missing, however, and as far as we know, no further studies have been performed. More studies on the occurrence of this SAA allelic variant are therefore warranted.

## 3. Systemic amyloidosis as a transmissible condition

In spite of a chronic inflammatory disease with persistent high plasma concentration of SAA, only some individuals develop AA-amyloidosis, even after a very long time. According to the above, one reason could be the expression of different SAA alleles. Another possibility may be *in vivo* fibril inhibitors, yet to be discovered. Another intriguing possibility is a seeding mechanism of AA- or other fibrils. There is no proof of this possibility in humans, but there is compelling evidence of this mechanism in the mouse. In many strains of mice, it is easy to induce a systemic AA-amyloidosis which is very similar to the human form. This can be done by chronic or repeated inflammatory challenge, either infectious or non-infectious. In the latter Freund's adjuvant, casein or silver nitrate is most often used. After a lag phase of several weeks or months, the animals develop AA-deposits first in the spleen, followed by the liver, kidneys and other organs. In 1966, Ranløv & Werdelin showed that transplantation of cells from an amyloidotic mouse to an inflamed animal drastically reduced the amyloid lag phase in the recipient (Werdelin & Ranløv 1966). In further experiments, Ranløv and co-workers could show that living cells are not necessary, but extracts from amyloid-containing tissues have the same properties (Hardt & Ranløv 1976). The active principle, later called ‘amyloid enhancing factor, AEF’, was for a long time elusive and the subject of a number of studies. There were many suggestions including glycoproteins and enzymes. Niewold *et al*. (1987) came to the conclusion that the AEF-activity was exerted by small fragments of amyloid fibrils. That fits well with our findings that the extract of purified mouse AA-amyloid is very potent and reduces the lag time drastically even when given at extremely low dose (Lundmark *et al*. 2002). In this way, murine AA-amyloidosis is transmissible but only when the recipient is prone to develop this disease. Although the mechanism is prion-like, there is a fundamental difference between prion diseases and other conditions characterized by amyloid in that, as far as we know, there must be an underlying abnormality in the latter, resulting in a sufficient concentration of an amyloid-prone protein. This deviation from normal can be over-expression of a protein, a point mutation resulting in amino acid substitution or deletion or abnormal cleavage of the fibril protein precursor, or more.

## 4. Cross-seeding as a mechanism in amyloidosis

Cross-seeding as used in this communication means that one biochemical form of fibrils induces aggregation of another protein to amyloid. Seeding *in vitro* has a high degree of specificity and substitution of one amino acid residue can profoundly affect seeding capacity (Krebs *et al*. 2004; O'Nuallian *et al*. 2004). Nevertheless, fibrils from Aβ peptide may seed amyloid formation from the unrelated peptide, islet amyloid polypeptide (IAPP; O'Nuallian *et al*. 2004), in spite of only approximately 25 per cent identity.

We were able to show that synthetic fibrils made from defined small peptides, corresponding to parts of known amyloid fibril proteins, reduce the lag time significantly in the murine AA-amyloidosis system (Ganowiak *et al*. 1994; Johan *et al*. 1998). In these experiments, small amounts of fibrils were injected intravenously in the tail vein of mice given an inflammatory challenge at the same time. These fibrils co-localized with the first amyloid deposits that appeared in the marginal zone of splenic lymph follicles. Also, small aggregates of synthetic fibrils were trapped in lung capillaries and these contained protein AA as well, which indicates a direct elongation of the synthetic materials by this protein, resulting in mixed fibrils (Johan *et al*. 1998). In nature, there are many examples of cross-beta-sheet fibrils with properties of amyloid, including *Bombyx mori* silk, Sup 35 from *Saccharomyces* and curli from *Escherichia coli*. All these also exert AEF properties in the murine AA-amyloidosis model (Lundmark *et al*. 2005). Finally, in collaboration with S. Zhang, MIT, Cambridge, MA, we have studied the effects of amyloid-like fibrils, made from non-natural short peptides intended for nano-technology, and found that these also can speed up the development of AA-amyloidosis in the same system (P. Westermark, S. Zhang & G. T. Westermark, unpublished results). However, in all these examples with synthetic peptides and naturally occurring amyloid-like fibrils, the lag phase was longer than when native murine AA-fibrils are used and, typically, a number of animals did not develop amyloidosis at all during the observation time. In this respect, the situation resembles the species barrier described for experimental transmission of prion diseases (Vanik *et al*. 2004; Scott *et al*. 2005).

## 5. Transmission of amyloidosis with native amyloid fibrils

Transmission of AA-amyloidosis to sensitive mice is efficient with a very low dose of material. This has also been seen with murine systemic apoliprotein A-II-derived amyloidosis (Ge *et al*. 2007). In addition, transmission of AA-amyloidosis has been found in hamster (Niewold *et al*. 1987), mink (Sørby *et al*. 2008) and duck (Benditt 1976). The latter two show that transmission is not restricted to rodents and there is no reason to believe that it could not happen in humans as well. It should be noted that transmission in mice is efficient not only by intravenous injection but also by the oral or nasal route (Lundmark *et al*. 2002). The risk of transmission is probably very low for human systemic amyloidoses, but the following patient story illustrates that this possibility cannot be completely ignored.

**Figure 2:**
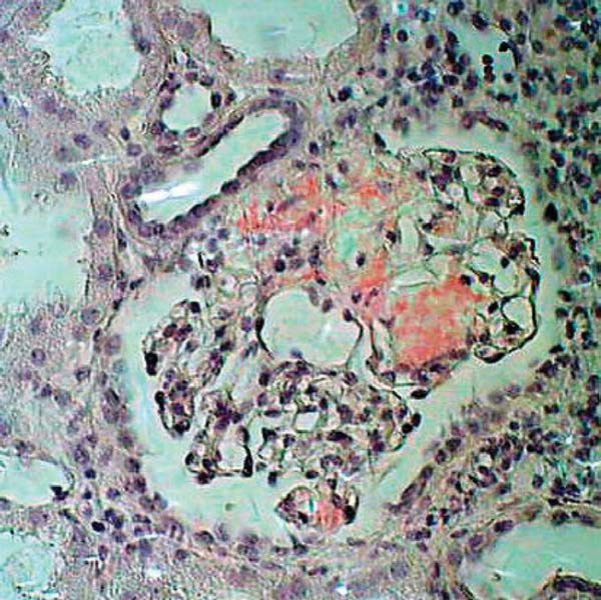
A glomerulus of an amyloidotic kidney, by mistake used for transplantation into a patient with renal insufficiency. Congo red in polarized light.

A 50-year-old woman underwent a renal cadaveric transplantation. The history of the donor was not quite well known and the kidney came from another European country. After transplantation, the concentration of creatinine in plasma did not decrease as expected. A renal biopsy was performed and that showed pronounced amyloid deposits in the glomeruli (figure 2). A subsequent biopsy verified this finding. Due to lack of material, no determination of amyloid type could be performed. The patient was lost during follow-up and the outcome is not known.

From this case history, it is obvious that transmission of amyloidosis between humans is possible. Given the low dose needed in the mouse model, contaminated surgical instruments may be a hazard; but no such case has yet been described.

Systemic AA-amyloidosis is not limited to humans and experimental animals, but spontaneously occurring disease is known from many mammalian and avian species (Zschiesche & Jakob 1989; Johnson *et al*. 1996). During the last years, it has become evident that systemic AA-amyloidosis is not rare in animals used as human food. Thus, Tojo *et al*. (2005) found a surprisingly high incidence of the condition in slaughtered cattle in Japan. It is also well known that AA-amyloidosis is prevalent in domestic ducks and geese (Brassard 1965; Zschiesche & Jakob 1989). In a recent study, Solomon *et al*. found AA-amyloid in commercially available duck liver and foie gras (Solomon *et al*. 2007), clearly demonstrating that this is something frequently eaten in certain parts of the world. Extract of this liver amyloid enhanced the development of amyloidosis in mice (Solomon *et al*. 2007). There are no reports of higher prevalence of the condition in countries with high consumption of duck and goose liver, such as France, but there are no good studies on the frequency of this disorder anywhere. In a recent amyloid induction study in mice, in which we used extracts of AA-amyloid from a number of different mammalian and one avian species, it was obvious that species barriers may be present. Only fibrils from one species enhanced amyloidosis in most mice (P. Westermark *et al*., unpublished results). Therefore, it is quite possible that goose and duck AA-amyloids are completely harmless for humans, but that has to be shown.

## 6. Seeding of localized amyloid forms

Amyloid deposits are a hallmark of Alzheimer's disease and type 2 diabetes and are believed to be important in the pathogenesis of the neuron and beta-cell death, respectively, in these two conditions. Typically, these deposits are widely spread as small particles in the brain and the islets of Langerhans. A question is then whether the fibrils form spontaneously at each point or if they start at one or a few sites and then spread by seeding. Given the properties of fibrils of the constituting peptides, Aβ and IAPP, to efficiently seed further fibril formation *in vitro*, it is most reasonable to believe that spreading occurs by seeding, although the routes are not fully understood. The same should certainly be true with the different systemic amyloidoses. We have found that islet amyloidosis is seedable *in vivo* by injection of a trace amount of preformed fibrils made of synthetic IAPP into transgenic mice that express human IAPP (G. T. Westermark *et al*., unpublished results). An important and yet not much studied question is whether one kind of amyloid can cross-seed and induce another form of amyloidosis. This possibility is of interest since small local amyloid deposits of different biochemical nature are very common and associated with ageing (Cornwell *et al*. 1995).

## 7. Conclusions

Much interest has been focused on kuru, and studies of this and closely related diseases have been absolutely crucial for our present understanding of transmissible encephalopathies and also amyloidosis (Prusiner *et al*. 1983; Gajdusek 1994). When kuru now is disappearing from Papua New Guinea (Collinge *et al*. 2006), other forms of amyloidosis should attract more interest in this country. This may be particularly true for AA-amyloidosis, which most likely still is a serious health problem in the Watut Valley and probably in other parts of the country. The possibility of a genetic predisposition for the disease as well as potential environmental factors, in addition to infections, for the development of this life-threatening condition will be important to study.
